# No Evidence That Analgesic Use after COVID-19 Vaccination Negatively Impacts Antibody Responses

**DOI:** 10.4049/immunohorizons.2300090

**Published:** 2023-12-12

**Authors:** Bonnie J. Lafleur, Lisa White, Michael D. Dake, Janko Z. Nikolich, Ryan Sprissler, Deepta Bhattacharya

**Affiliations:** * BIO5 Institute, University of Arizona, Tucson, AZ; † Department of Pharmacy Practice and Science, R. Ken Coit College of Pharmacy, University of Arizona, Tucson, AZ; ‡ Office of the Senior Vice-President for Health Sciences, University of Arizona, Tucson, AZ; § Department of Immunobiology, University of Arizona College of Medicine, Tucson, AZ; ¶ University of Arizona Center on Aging, University of Arizona College of Medicine, Tucson, AZ; ‖ University of Arizona Genomics Core and the Arizona Research Labs, University of Arizona Genetics Core, University of Arizona, Tucson, AZ; # Department of Surgery, University of Arizona College of Medicine, Tucson, AZ

## Abstract

Uptake of mRNA vaccines, especially booster immunizations, against COVID-19 has been lower than hoped, perhaps in part due to their reactogenicity. Analgesics might alleviate symptoms associated with vaccination, but they might also impact immune responses. We semiquantitatively measured Ab responses following COVID-19 vaccination in 2354 human participants surveyed about analgesic use after vaccination. Participants who used nonsteroidal anti-inflammatory drugs or acetaminophen after vaccination showed elevated Ab levels against the receptor-binding domain of Spike protein relative to those who did not use analgesics. This pattern was observed for both mRNA-1273 and BNT162b2 and across age groups. Participants who used analgesics more frequently reported fatigue, muscle aches, and headaches than did those who did not use painkillers. Among participants who reported these symptoms, we observed no statistically significant differences in Ab levels irrespective of analgesic use. These data suggest that elevated Ab levels are associated with symptoms and inflammatory processes rather than painkiller use per se. Taken together, we find no evidence that analgesic use reduces Ab responses after COVID-19 vaccination. Recommendation of their use to alleviate symptoms might improve uptake of booster immunizations.

## Introduction

Messenger RNA vaccines against SARS-CoV-2, the virus that causes COVID-19, showed high efficacy against symptomatic illness caused by the ancestral strain (1, 2). Yet in part due to viral evolution and escape from neutralizing Abs and in part due to waning of protective Ab production, the initial effectiveness of these vaccines has decreased from their peaks, especially against mild symptomatic infections (3). In response, vaccines updated to more closely match the circulating lineages have been made available in hopes of restoring high levels of protection against SARS-CoV-2 infections and COVID-19. Studies of neutralizing Ab levels and real-world effectiveness showed the benefit of bivalent boosters updated to match the BA.4-5 lineages at protecting against variants of concern (Refs. 4–10, S. Chalkias, S., J. Whatley, F. Eder, B. Essink, S. Khetan, P. Bradley, A. Brosz, N. McGhee, J.E. Tomassini, X. Chen, et al., manuscript posted on medRxiv, DOI: 10.1101/2022.12.11.22283166, and J. Zou, C. Kurhade, S. Patel, N. Kitchin, K. Tompkins, M. Cutler, D. Cooper, Q. Yang, H. Cai, A. Muik, et al., manuscript posted on bioRxiv, DOI: 10.1101/2022.11.17.516898). Yet uptake was poor, as only ∼30% of eligible adults in the United States received the bivalent booster (11). Uptake is also likely to be low for the latest monovalent booster that has been updated to match the XBB1.5 lineage. New public campaigns and strategies are needed to improve the uptake over what was observed previously.

The reactogenicity of mRNA vaccines might negatively impact willingness to receive booster immunizations (12). Although serious side effects following vaccination are rare, mRNA vaccines frequently lead to mild local and systemic adverse events such as injection site pain, lymphadenopathy, myalgia, and fever (13, 14). Over-the-counter analgesics after vaccination might mitigate some of these mild adverse events, but animal models of SARS-CoV-2 infection have shown that nonsteroidal anti-inflammatory drugs (NSAIDs) substantially reduce antiviral Ab responses (15). It remains unclear whether these same inhibitory effects are seen in humans after mRNA vaccination and how different classes of painkillers impact the Ab response. Several prior studies have suggested minimal or even positive correlations of antipyretic use with Ab responses (16–18). Further analysis of their impact, independent of symptoms and inflammation, as well as subgroup analyses on variables such as age, vaccine type, and the class of analgesics, would be valuable to help interpret these findings.

## Materials and Methods

### Human subjects

All human subject work was approved by the University of Arizona Institutional Review Board and was conducted in accordance with all federal, state, and local regulations and guidelines under protocols 1510182734 and 1410545697A048. Subjects <18 y of age were excluded. Subjects were recruited via public announcement and Web site registration as part of the University of Arizona Ab testing initiative. Following Web site registration, subjects were ascertained to be afebrile and without COVID-19 symptoms based on a questionnaire, were consented, and bled. Blood was centrifuged at multiple sites across Arizona. For all subjects, venous blood was obtained by venipuncture into SST Vacutainer tubes (Becton Dickinson, Sunnyvale, CA, catalog no. 367988), and serum was separated by centrifugation at 1200 rpm and sent to the central processing laboratory within 4 h.

### ELISAs

**FIGURE 1.**
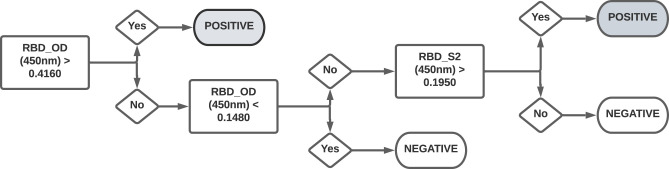
Workflow for diagnostic serological testing. Serum samples were first tested for Abs against RBD. OD_450_ values >0.4160 were considered as seropositive, whereas values <0.1480 were considered seronegative. Samples with OD_450_ values between 0.1480 and 0.4160 were further tested for Abs against S2. Of these samples, OD_450_ values >0.1950 were considered seropositive and all others were considered seronegative.

Mammalian RBD was purchased from GenScript (catalog no. Z03483). S2 of the Spike protein was purchased from Sino Biological (catalog no. 40590-V08B). ELISA was performed as described (19). Plates were blocked with 1% nonfat dehydrated milk extract (Santa Cruz Biotechnology, catalog no. sc-2325) in sterile PBS (Fisher Scientific/HyClone PBS, catalog no. SH2035) for 1 h, washed with PBS containing 0.05% Tween 20, and overlaid with a 1:40 dilution of serum for 60 min. Plates were then washed and incubated for 1 h in 1% PBS and milk containing an anti-human pan-Ig HRP-conjugated Ab (Jackson ImmunoResearch, catalog no. 109-035-064) at a concentration of 1:2000 for 1 h. Plates were washed with PBS with Tween 20 solution followed by a PBS wash. To develop, plates were incubated in tetramethylbenzidine prior to quenching with 2 N H_2_SO_4_. Plates were read for 450 nm absorbance on CLARIOstar Plus from BMG Labtech. Samples with OD_630_ values >0.05 were rerun. Every plate contained at least 32 seronegative controls and either CR3022 or HM3128 (Creative Diagnostics) mAbs as a positive control for RBD or S2, respectively. This assay has received emergency use authorization from the U.S. Food and Drug Administration (ID 201116). The diagnostic workflow is shown in Fig. 1.

### Statistical analysis

Two-sided *t* test statistics within one-way ANOVA were used to compare pairwise receptor-binding domain (RBD) ELISA OD_450_ means between pain reliever groups at a significance level of 0.05. Logistic regression, using likelihood ratio χ^2^ test statistics, with a significance level of 0.05, were used to compare the proportion of participants experiencing symptoms by pain reliever groups. Comparisons of symptoms by seropositivity and seronegativity are descriptive, owing to the small sample size of the seronegative participants. All analysis was performed using the R programming language (version 4.3.1).

## Results

**FIGURE 2.**
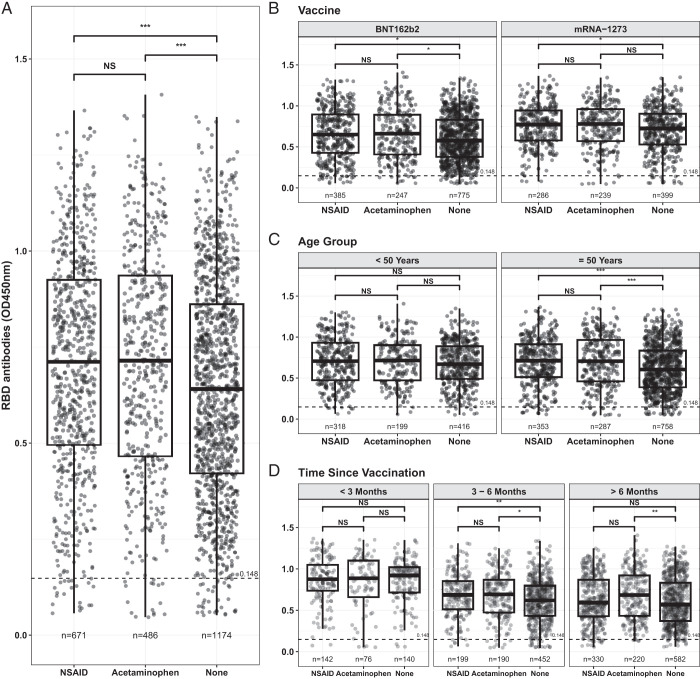
RBD-specific Ab levels postvaccination. (**A–C**) Pairwise means of RBD ELISA OD_450_ by pain reliever group (A), additionally stratified by vaccine (B) and age (C) were compared using one-way analysis variance. (**D**) Time since vaccination. Horizontal dashed line denotes the cut-off for seronegative status. Pairwise differences were compared using a two-sample *t* test with *p* value adjustment using the Tukey honestly significant difference. Total numbers of individuals tested in each category are shown below each plot. *p < 0.05, **p < 0.01, ***p < 0.001.

We examined survey results for analgesic use from March 15, 2021 through March 22, 2022 that included 2354 vaccinated individuals who were part of a large statewide Ab testing initiative run by the University of Arizona (Table I). Abs against the SARS-CoV-2 Spike RBD, self-reported vaccination information, and reported analgesic use within 48 h after either of the two-dose mRNA COVID-19 vaccines (mRNA-1273 or BNT162b2) were analyzed. A one-way ANOVA, using the *t* test statistic, showed statistically significantly lower RBD-specific Ab levels in those who did not take an analgesic (*n* = 1184) relative to those who took either NSAIDs or acetaminophen (Fig. 2A, *p* = 0.0001 for both). There was no statistically significant difference detected between the NSAID (*n* = 679) and acetaminophen (*n* = 491) analgesic groups (Fig. 2A, *p* = 0.9332). Average RBD-specific Ab levels were statistically higher for NSAID users compared with those not using pain medication for both BNT162b2 and mRNA-1273 (Fig. 2B, *p* < 0.05). Acetaminophen users also showed higher RBD-specific Ab levels than those not taking analgesics for individuals who received mRNA-1273 (Fig. 2B, *p* < 0.05). Elevated RBD-specific Ab levels were statistically higher in individuals ≥50 y old who took either NSAIDs or acetaminophen relative to those who took no analgesics (Fig. 2C, *p* < 0.001); these differences were not apparent in adults <50 y of age (Fig. 2C). At <3 mo postvaccination, Ab levels in those who used analgesics were not statistically significantly greater than those who did not use painkillers, but these differences became apparent afterward (Fig. 2D). For reasons that are unclear, and therefore results are not shown, those who did not answer the painkiller questionnaire (*n* = 1489) had statistically significantly higher RBD levels than did those who did respond to the survey.

**Table I.** Demographics information

	NSAID*^a^* (*n* = 679)	Acetaminophen*^b^* (*n* = 491)	No Pain Medication (*n* = 1184)	Overall (*n* = 2354)
Age (y)				
Mean (SD)	50.4 (15.9)	53.4 (15.4)	55.1 (16.6)	53.4 (16.3)
Median [minimum, maximum]	51.2 [18.2, 99.0]	54.6 [18.0, 91.5]	57.9 [18.0, 92.6]	54.9 [18.0, 99.0]
Sex at birth				
Female	488 (71.9%)	367 (74.7%)	693 (58.5%)	1548 (65.8%)
Male	185 (27.2%)	123 (25.1%)	491 (41.5%)	799 (33.9%)
Missing	6 (0.9%)	1 (0.2%)	0 (0%)	7 (0.3%)
Race/ethnicity				
Hispanic or Latino	108 (15.9%)	98 (20.0%)	173 (14.6%)	379 (16.1%)
Non-Hispanic black	11 (1.6%)	6 (1.2%)	11 (0.9%)	28 (1.2%)
Non-Hispanic other	64 (9.4%)	46 (9.4%)	119 (10.1%)	229 (9.7%)
Non-Hispanic white	496 (73.0%)	341 (69.5%)	881 (74.4%)	1718 (73.0%)
RBD (OD)				
Mean (SD)	0.702 (0.292)	0.708 (0.299)	0.643 (0.285)	0.673 (0.291)
Median [minimum, maximum]	0.714 [0.0570, 1.37]	0.717 [0.0470, 1.41]	0.641 [0.0520, 1.35]	0.676 [0.0470, 1.41]
Serostatus				
Positive	644 (94.8%)	463 (94.3%)	1121 (94.7%)	2228 (94.6%)
Negative	35 (5.2%)	28 (5.7%)	63 (5.3%)	126 (5.4%)

a For example, ibuprofen, naproxen, aspirin.

b For example, Tylenol.

**FIGURE 3.**
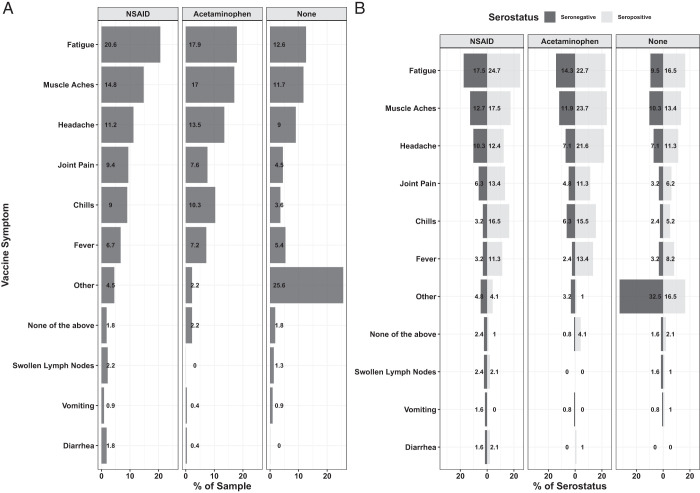
Rates of symptoms postvaccination. (**A** and **B**) Percent of participants experiencing each symptom (A) as well as stratified by serostatus (B). Logistic regression analysis was used to examine the difference between the proportion of symptoms and pain reliever use. There was a statistically significant difference in the proportion of participants experiencing fatigue and muscle aches between NSAID users and those not taking pain medication (*p* < 0.0001 for both). We also observed a statistically higher proportion of headaches reported by NSAID users, compared with those not taking pain medication and lower than those taking acetaminophen (*p* < 0.0088). Numbers of individuals in each category are shown in Table II.

Twenty-eight percent of the participants who completed the survey took NSAID pain relievers, compared with 21% taking acetaminophen and 50% not taking any pain medication. The most common vaccine-induced side effects were fatigue, muscle aches, and headaches, and were higher in those taking NSAIDs and acetaminophen compared with those not taking any pain relievers (Fig. 3A, Table II). Logistic regression analyses showed that there was a statistically significantly higher proportion of participants taking pain medication who experienced fatigue and muscle aches, particularly between those taking NSAIDs and those not taking pain medication (*p* < 0.0001 for both vaccine side effects). We also observed a statistically higher proportion of NSAID users reporting headaches, compared with those not taking pain medication, and lower than those taking acetaminophen (*p* < 0.0088). There were other side effects that were not captured on the survey (denoted as “other”), which were most common among those not taking any pain medication (Fig. 3A, 23.8%).

**Table II.** Number and percent of participants experiencing symptoms by NSAID use and serostatus

	NSAID, No. (%)		Acetaminophen, No. (%)		No Pain Medication	
Symptoms	Seronegative	Seropositive	Seronegative	Seropositive	Seronegative	Seropositive
Fatigue	22 (17.5)	24 (24.7)	18 (14.3)	22 (22.7)	12 (9.5)	16 (16.5)
Muscle aches	16 (12.7)	17 (17.5)	15 (11.9)	23 (23.7)	13 (10.3)	13 (13.4)
Headache	13 (10.3)	12 (12.4)	9 (7.1)	21 (21.6)	9 (7.1)	11 (11.3)
Joint pain	8 (6.3)	13 (13.4)	6 (4.8)	11 (11.3)	4 (3.2)	6 (6.2)
Chills	4 (3.2)	16 (16.5)	8 (6.3)	15 (15.5)	3 (2.4)	5 (5.2)
Fever	4 (3.2)	11 (11.3)	3 (2.4)	13 (13.4)	4 (3.2)	8 (8.2)
Other	6 (4.8)	4 (4.1)	4 (3.2)	1 (1)	41 (32.5)	16 (16.5)
None of the above	3 (2.4)	1 (1)	1 (0.8)	4 (4.1)	2 (1.6)	2 (2.1)
Swollen lymph nodes	3 (2.4)	2 (2.1)	0 (0)	0 (0)	2 (1.6)	1 (1)
Vomiting	2 (1.6)	0 (0)	1 (0.8)	0 (0)	1 (0.8)	1 (1)
Diarrhea	2 (1.6)	2 (2.1)	0 (0)	1 (1)	0 (0)	0 (0)

**FIGURE 4.**
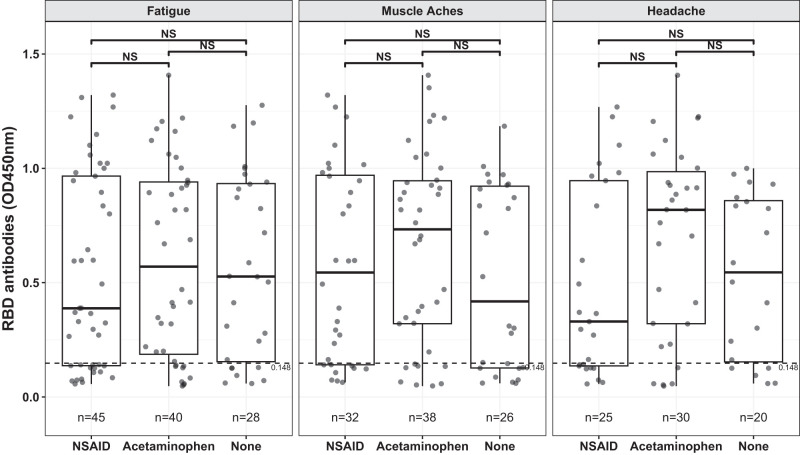
RBD-specific Ab levels stratified by symptoms and analgesic use. Pairwise means of RBD ELISA OD_450_ by pain reliever group for the top three vaccine induced symptoms are shown. Horizontal dashed line denotes the cut-off for seronegative status. Pairwise differences were compared using a two-sample *t* test with *p* value adjustment using the Tukey honestly significant difference. Total numbers of individuals tested in each category are shown below each plot.

Although 95% of participants were scored as diagnostically seropositive after vaccination, a small fraction of individuals (126/2354) fell below the seropositive threshold, based on RBD and S2 Ab levels (see *Materials and Methods* for details). The percentage of participants reporting fatigue, muscle aches, headaches, joint pain, chills, and/or fever were greater in those who tested seropositive relative to those who were diagnostically seronegative (Fig. 3B, Table II). To further examine these relationships, we compared Ab levels in the subsets of participants who reported fatigue, muscle aches, and/or headaches across analgesic use categories (Fig. 4). We observed no statistically significant differences in RBD-specific Ab levels across different analgesic use categories when participants were matched for these symptoms (Fig. 4).

## Discussion

Despite high effectiveness against symptomatic infections and severe disease (8–10, 20), the uptake of both the original mRNA COVID-19 vaccines and updated boosters in the United States has been lower than desired (12). There are many reasons for this low uptake, but one factor is the reactogenicity of the mRNA vaccines (12). For adenovirus-based COVID-19 vaccines, antipyretic use does alleviate symptoms (21), and may do so as well for mRNA vaccines. Widespread recommendation of their use after mRNA vaccination may thus improve booster uptake.

Unlike animal models of SARS-CoV-2 infections (15), several prior studies observed no detriment to postvaccination Ab responses by analgesic use, but differed on whether their use was in fact associated with elevated titers (16–18). A causal positive relationship between analgesic use and Ab levels would be unexpected, and indeed we found no evidence to support such a relationship. Instead, several other studies reported positive correlations between postvaccination symptoms and Ab levels (Refs. 22–24 and E.G. Dutcher, E.S. Epel, A.E. Mason, F.M. Hecht, J.E. Robinson, S.S. Drury, and A.A. Prather, manuscript posted on medRxiv, DOI: 10.1101/2023.09.26.23296186). The inflammation that causes these symptoms seems more likely than analgesics to be causally related to elevated Ab levels. Because analgesic use is more common in those who experience symptoms, their association with Ab titers might simply be a correlative marker of these adverse events. By examining each of these parameters in a relatively large cohort, our data confirmed that analgesic use and certain symptoms correlate with elevated Ab levels. This association held across mRNA vaccines and became more apparent over time. However, analgesic use did not clearly impact Ab responses independently of symptoms, positively or negatively.

Prior studies on SARS-CoV-2 infections showed clear positive associations of COVID-19 disease severity and inflammation with Ab levels (25), although the quality of the Abs might be negatively impacted with excessive inflammation (26). It may be that for vaccines, inflammation and associated side effects are also causally linked to Ab titers. As one possible mechanistic explanation, certain HLA haplotypes are associated with adverse events after vaccination (27). These haplotypes may also be associated with improved CD4^+^ T cell help for B cell responses and resultant Ab levels.

Although our data support widespread recommendations of analgesic use to mitigate post–COVID-19 vaccination side effects, current public health campaigns may encourage contemporaneous receipt of influenza and respiratory syncytial virus vaccines. Although the impact of analgesics on influenza vaccine responses has been reported to be minimal (28), their impact on respiratory syncytial virus vaccine responses remains unknown. Our study does have some additional limitations in that we did not examine other parameters of immunity, such as memory B and T cells, nor did we examine the impact of analgesics that were taken prior to vaccination. Moreover, although our semiquantitative RBD Ab assays correlate reasonably well with neutralizing Abs (19), we did not directly measure Ab quality or function in this study. Finally, because our study was not a prospective randomized trial and relies on self-reporting of analgesic use and symptoms, causal relationships between these variables and Ab levels are difficult to infer.
